# Correlation of social cognition and neurocognition on psychotic outcome: a naturalistic follow-up study of subjects with attenuated psychosis syndrome

**DOI:** 10.1038/srep35017

**Published:** 2016-10-10

**Authors:** TianHong Zhang, HuiRu Cui, YingYing Tang, LiHua Xu, HuiJun Li, YanYan Wei, XiaoHua Liu, Annabelle Chow, ChunBo Li, KaiDa Jiang, ZePing Xiao, JiJun Wang

**Affiliations:** 1 Shanghai Mental Health Center, Shanghai Jiaotong University School of Medicine, Shanghai Key Laboratory of Psychotic Disorders, Shanghai 200030, P. R. China; 2 Florida A & M University, Department of Psychology, Tallahassee, Florida 32307, USA; 3 Changi General Hospital, Department of Psychological Medicine, Singapore; 4 Bio-X Institutes, Key Laboratory for the Genetics of Developmental and Neuropsychiatric Disorders (Ministry of Education), Shanghai, China.

## Abstract

Neurocognitive decline has been observed in patients with psychosis as well as attenuated psychosis syndrome (APS). We tested the hypothesis that APS increases dependence on neurocognition during the interpretation of others’ mental states and that a combination index of Theory of Mind (ToM) and neurocognition improves the predictive accuracy of psychosis conversion. A sample of 83 APS individuals and 90 healthy controls (HC) were assessed by comprehensive cognitive tests. The cohort also completed a one-year follow-up. In the APS group, ToM was associated with an apparent increase in neurocognition, but this trend was not evident in the HC group. Using the new index of combined neurocognition and ToM scores, the sensitivity for predicting psychosis-proneness was 75% and the specificity was 69%. Our data suggest that the correlations between ToM function and neurocognition in APS subjects were stronger than those in healthy controls. A composite index of neurocognition and ToM could improve the predictive validity of a future conversion to psychosis.

There is now substantial evidence for cognitive deficits in individuals with psychosis^1^,^2^,^3^, which is particularly related to their functional outcomes^4^. We previously reported that psychosis is characterized by a pervasive impairment in cognition during all stages of illness^5^. Furthermore, several models have further examined patients with psychosis and have implicated an association between cognitive impairment and negative symptoms^6^, cost of care^7^, as well as self-reported real world functioning^4^. However, once psychosis occurred, cognitive assessment was affected by a substantial variance, which was not accounted for by the identified factors. Cognition studies focusing on the post-onset stage of psychosis are typically impacted by the effects of full blown psychotic symptoms (which can vary in severity and affect test compliance), long periods with lack of treatment, stigma-related issues (e.g., social exclusion and isolation), medication and other mass interventions, which may represent potential confounds. One approach to minimizing these confounds is to focus on cognitive function during the pre-onset stage of psychosis.

Attenuated psychosis syndrome (APS) is a new disorder proposed in the DSM-V (the Diagnostic and Statistical Manual of Mental Disorders, 5th Edition). APS is conceptualized as a transitional clinical stage in which the individual suffers from recent psychotic experiences, but realizes that these experiences are not tethered to reality^8^. In turn, this stage can be clearly interpreted as the affected individual having some degree of insight, compared to full blown psychosis. APS criteria emphasizes a recent onset, self-limited psychotic symptoms and the seeking of professional help. Our previous studies^9^,^10^ have reported APS individuals are more likely to look for help using their own initiative, to be drug naive, to not be severely withdrawn from social environment, and to be cooperative with cognitive tests and the follow-up process. Therefore, there are obvious advantages to performing cognitive assessments in individuals with APS, which would provide more direct, effective and “clean” evidence regarding the cognitive model and its impact on the progression of psychosis.

Since the level of insight is a core feature of psychotic development, theory of mind (ToM) is one of the first cognitive impairments in individuals with APS. These functional deficits in ToM may underlie some issues related to maintaining insight during psychotic development^11^. ToM contributes to this process and may be the best cognitive mediator of psychotic development^12^. A functional deficit in ToM is defined as an underlying difficulty in interpreting the mental states of others, included 2 facets of functioning: the decoding mental states (DMS) and the reasoning mental states (RMS)^13^. The first facet is related to social perception, which relies on the immediate response to available perceptual information. The second facet is related to the cognitive inference about others’ situation, which is considered to be a traditional aspect of ToM and usually requires higher-order cognitive function. ToM processes through networks rather than single structures and the 2 facets are closely connected; this distinction has been supported by fMRI and lesion studies. For example, the amygdala and other medial temporal lobe regions play a crucial role in DMS, while the prefrontal cortex is more involved in RMS^14^.

Given that mild impairments in both neurocognition and ToM have been reported in the APS population^15^,^16^, it is important to understand if ToM is affected by neurocognition and further, if it contributes to the progression of psychosis. Neurocognition has been positively linked to ToM in previous studies, but these studies were limited by small sample size, cross-sectional design, medication confounds, unadjusted correlation analyses and the lack of proper controls. Further, considerable heterogeneity has been observed in these studies^17^,^18^.Whether ToM functioning is multi-dimensional and whether ToM functioning contains different routes involving various neural substrates is not completely understood. However, these results mainly demonstrate that ToM relies on neurocognition as a ‘necessary, but not sufficient’ prerequisite in patients with schizophrenia. Consequently, a change in the cognitive model for the processing and interaction between ToM and neurocognition could be important in characterizing prodromal psychosis. Furthermore, it could even be a significant predictive factor for the subsequent conversion to psychosis.

Based on previous studies focusing on the relationship between ToM and neurocognition, we hypothesized that the characteristic of cognitive correlation is different in individuals with APS compared to healthy controls. Specifically, we hypothesized that ToM deficits among APS subjects would increase their correlation with neurocognition when interpreting others’ mental states. Given the proven power of cognition in predicting psychotic outcomes, if the increased correlations are correct, either neurocognition or ToM performance alone may not be the best predictor of APS outcomes. Therefore, the combination index of ToM and neurocognition could improve the predictive accuracy for individuals who are at real risk for developing psychosis.

## Methods

### Subjects and Procedures

The study was conducted following the tenets of the Helsinki Declaration and approved by the Research Ethics Committee of the Shanghai Mental Health Center (SMHC) in 2012. All participants gave written informed consent at the recruitment stage of the study. The attenuated positive symptom (APS) group was comprised of 83 subjects with attenuated positive symptom syndrome (APSS) who fulfilled the diagnostic criteria of SIPS/SOPS (Structured Interview for Prodromal Symptoms/Scale of Prodromal Syndromes)^19^. Subjects were recruited using both a clinic-wide questionnaire screening and clinician referrals at the SPCC (Shanghai Psychotherapy and Psychological Counseling Center) of SMHC. All subjects had their first visit to a mental health service and were antipsychotic naïve at baseline. Subjects with a history of substance abuse, severe somatic disease, dementia or other psychoses with unrelated cognitive deficits were excluded. One hundred clinical high risk (CHR) subjects were invited to undergo cognitive testing. In the current study, 83 APS subjects volunteered to complete all the tests at baseline. APS status was confirmed by 2 psychiatrists using SIPS/SOPS. Subjects were aware of the study plan for a 1 year follow-up.

Healthy controls (HC) were recruited from the local school and community through an internet advertisement. Individuals in the HC group who completed a face-to-face interview and cognitive tests received ¥100 (approximately $15) as compensation for their time. The SIPS/SOPS and MINI (Mini International Neuropsychiatric Interview)^20^ were administered by a senior psychiatrist to exclude any prodromal psychotic symptoms or other mental disorders. Participants who reported a family history of mental disorders were excluded. The demographic background (e.g., age, gender, marital status, and years of education) were closely matched to the APS group. One hundred HC subjects were invited to participate; 90 individuals completed the tests. Written informed consent was issued to all subjects. Participants who were younger than 18 years of age had their informed consent forms co-signed by their parents.

Follow-up examinations were carried out 1 year after baseline. A conversion to psychosis was determined using the criteria of POPS (Presence of Psychotic Symptoms in SIPS/SOPS)^21^. At least one psychotic level symptom (rated a “6” on at least 1 of the 5 positive symptoms) was required for the fulfillment of POPS criteria, with either sufficient symptom frequency and duration or at a level that was disorganized or dangerous. The simplified SIPS interview (only positive symptoms in SIPS/SOPS were “inquired” and “anchored”) was conducted either face-to-face or with a phone call with APS subjects at a 1year interval to determine the major outcome (e.g., conversion to psychosis). If APS subjects felt their previous symptoms were worse or their family members observed a deterioration, a re-assessment was conducted at any time before the scheduled follow-up point.

### Instruments

#### Clinical Assessment

The SIPS/SOPS was used at study entry and follow-up. The 19-item SIPS assessed prodromal positive symptoms and a range of other potentially prodromal phenomena (e.g., negative, disorganized and general symptoms). Symptoms were rated on a 0–6 scale, with scores of 3–5 in positive symptoms indicating attenuated psychotic symptoms. To fulfill the APSS criteria, those attenuated positive symptoms must have either begun in the past year or increased in intensity by at least one rating point. Meanwhile, these symptoms must have occurred at the current intensity level for an average frequency of at least once per week in the past month. The assessments were conducted by a senior psychiatrist who was certified on the SIPS at a Yale University-sponsored SIPS/SOPS training. With permission from the Yale authors, the Chinese version of the SIPS/SOPS (Version 5) was translated by our team and tested for its reliability and validity in a Chinese clinical population^22^,^23^.The MINI interview was conducted to screen for the majority of mental disorder categories (17 major psychiatric disorders based on the criteria of the DSM-IV), and also covered different periods, including the past, present, and lifespan.

#### The MCCB (MATRICS Consensus Cognitive Battery)

The Chinese version of the MCCB^24^ was administered with the standardized guidelines provided in the test manual to assess neurocognition. Consistent with the original version of the MCCB^25^,^26^, the Chinese version covered the following 7 domains: 1) speed of processing (SP, the Part A of Trail Making Test, the Symbol Coding Test and the Category Fluency Test), 2) attention/vigilance (AV, the Continuous Performance Test-Identical Pairs), 3) working memory (WM, the spatial span of the Wechsler Memory Scale-III), 4) verbal learning (VeL, the Revised Hopkins Verbal Learning Test), 5) visual learning (ViL, the Revised Brief Visuospatial Memory Test), 6) reasoning and problem solving (RPS, the Neuropsychological Assessment Battery: Mazes), and 7) social cognition. The social cognition domain was not applied in this study because the test is not applicable for subjects under age of 18 years, and did not fit the neurocognition category.

We inputted data twice (double data entry) into the MCCB computer program and converted the raw scores to T-scores for the 7 domains and a composite T-score (OCS, Overall Composite Neurocognitive Score). Age- and sex-corrected Chinese norms were used according to the guidelines outlined in the Chinese version of the MCCB manual. The Chinese version has also been used to identify cognitive deficits in different populations, especially for patients with psychosis, with a test-retest reliability of subtests ranging from 0.73 to 0.94^24^.

#### ToM tests

ToM functioning is typically assessed using 2 different aspects^27^, DMS and RMS. The ToM sub-process of DMS is proposed to be the perception of social information (e.g., eye expression) from a second person. In contrast, RMS is proposed to be the integration of social cues in order to infer the belief of a second person to a third person. We assumed that DMS is a basic sub-process of ToM functioning, and accordingly, RMS is an advanced sub-process, which is even more complicated.

DMS was measured by the Reading-the-Mind-in-the-Eyes Test (RMET). The RMET score was derived from the summation of the percent correct responses to a static photograph of the eye region. Subjects were asked to select 1of 4 words that most appropriately described what the person in the photograph maybe thinking or feeling; there was 1 correct response and 3 distracters. Both the English (RMET-EN) and the Chinese (RMET-CH) version of the RMET were used in this study. The RMET-EN, designed by Baron-Cohen^28^, consisted of 36 photographs, while the RMET-CH, designed by Y.G. Wang^29^, consisted of 34 photographs of the eye region of Asian individuals, consistent with RMET-EN material.

RMD was evaluated with the social faux-pas (FP) task^30^, which required the subject to identify whether someone had said something wrong or had upset others in a variety of social situations. The test consisted of 20 verbal stories, including 10 with a social FP (FP-F) and 10 without a FP (FP-T). In situations where the subject identified a FP, additional questions were asked to assess the subject’s understanding of the FP. Minor modifications were made for social contexts in Chinese culture, according to a preliminary survey using the FP prior to this study^31^. The Chinese version was very similar to the English version, with hits or clues remaining unchanged.

To standardize ToM test procedures, we developed a computer program based on the E-prime 2.0 software, integrating the RMET and FP tests for this study. For RMET, each pair of eyes, along with 4 words, was presented on a 19-inch monitor. Subjects could make their decision without a time limit by pressing 1 of the 4 keys, which corresponded to the 4 words on the screen. For FP, the stories were shown on a computer screen and narrated in the same tone of voice. Participants then answered questions based on each story; the story remained on the screen to reduce the subject’s memory load.

### Data Analysis

Statistical analysis was performed with SPSS version 16.0 (SPSS, Inc., Chicago, IL, USA). Parametric data from the APS and HC groups were compared using independent sample t-tests. Discrete variables were compared using a chi-squared test. Analyses of covariance (ANCOVA) were performed to examine differences between the groups in 1) the MCCB domains (SP, AV, WM, VeL, ViL, RPS, OCS) with age and years of education entered as covariates; and 2) the FP and RMET domains (FP, FP-F, FP-T, RMET-CH, RMET-EN) with age, years of education, SP, AV, WM, VeL, ViL, RPS, and OCS entered as covariates. The relationship between ToM performance in the domains of MCCB scores were characterized using linear trend lines. A set of regression lines were drawn to highlight the trend of APS subjects compared to HC subjects. The decline slopes of trend lines were compared between APS and HC by SPSS [Detailed method can be found at http://www.ats.ucla.edu/stat/SPSS/faq/compreg2.htm]. We proposed that if the APS slopes are significantly different (greater) from the HC slopes, this may indicate a greater degree of relationship in APS than in HC. We compared the regression coefficients of APS with HC to test the null hypothesis Ho: *β*_APS_ = *β*_HC_, where *β*_APS_ is the regression coefficient for APS, and *β*_HC_ is the regression coefficient for HCs. The *p*-values were reported for the *β* weights (*p*_*β*_) and the differences between *β* weights (*p*_(*β*, APS vs. HC)_, and for slopes (coefficients) comparison between APS and HC). We controlled the family-wise error (FWE) at the 0.0024 (*p* < 0.05/21) level using a Bonferroni correction, to select neurocognition domains truly associated with social cognition domains (*p < 0.0024). The equations and associated *R*^*2*^ value (uncorrected *R* square values) were calculated by linear regression function with default parameters in SPSS. Correlation analyses were performed to evaluate correlations between ToM test scores and MCCB scores within the APS and HC groups.

To evaluate the predictive value of the cognitive domains for the conversion to psychosis, we performed a binary logistic regression analysis (forward method, conditional, entry: 0.05, removal: 0.1) to explore which domains at baseline best predicted converters and non-converters. The regression models included 6 neurocognitive domains and 2 ToM subtests (FP, RMET), which were analysed separately as independent variables. We further evaluated the predictive values (e.g., sensitivity and specificity) of the psychosis-proneness index, which was derived from a combination of ViL and FP scores according to the regression model. Receiver operating characteristic (ROC) analysis was used to test whether the combined neurocognitive and ToM index allows distinguishing between converters and non-converters. The predictive value of the combined index and cognitive domains alone was determined according to the area under the ROC curve (AUC) as follows: non-predictive, AUC < 0.5; less predictive, 0.5 < AUC < 0.7; moderately predictive, 0.7 < AUC < 0.9; highly predictive, 0.9 < AUC < 1^32^.

## Results

### Demographics and Clinical Characteristics

Demographics and clinical characteristics are presented in Table 1. There was no significant difference in the proportion of males to females, age or educational levels between the APS and HC groups. In the APS group, the most frequent positive symptoms were suspiciousness and unusual thoughts. The mean time between symptom onset and professional help-seeking was approximately 5 months.

### Cognitive Performance comparisons in the HC and APS groups

Comparisons of unadjusted mean cognitive performance scores revealed a significantly higher score for all cognitive domains in the HC group compared to APS subjects (Table 2). Age-education-adjusted mean scores were also significantly different in the cognitive domains between the 2 groups. Age-education-neurocognition-adjusted mean scores for ToM tests were significantly lower in the APS group compared to the HC group, but these differences were relatively smaller after adjusting for neurocognition.

### Correlations between ToM and Neurocognition Performance

**Figure 1:**
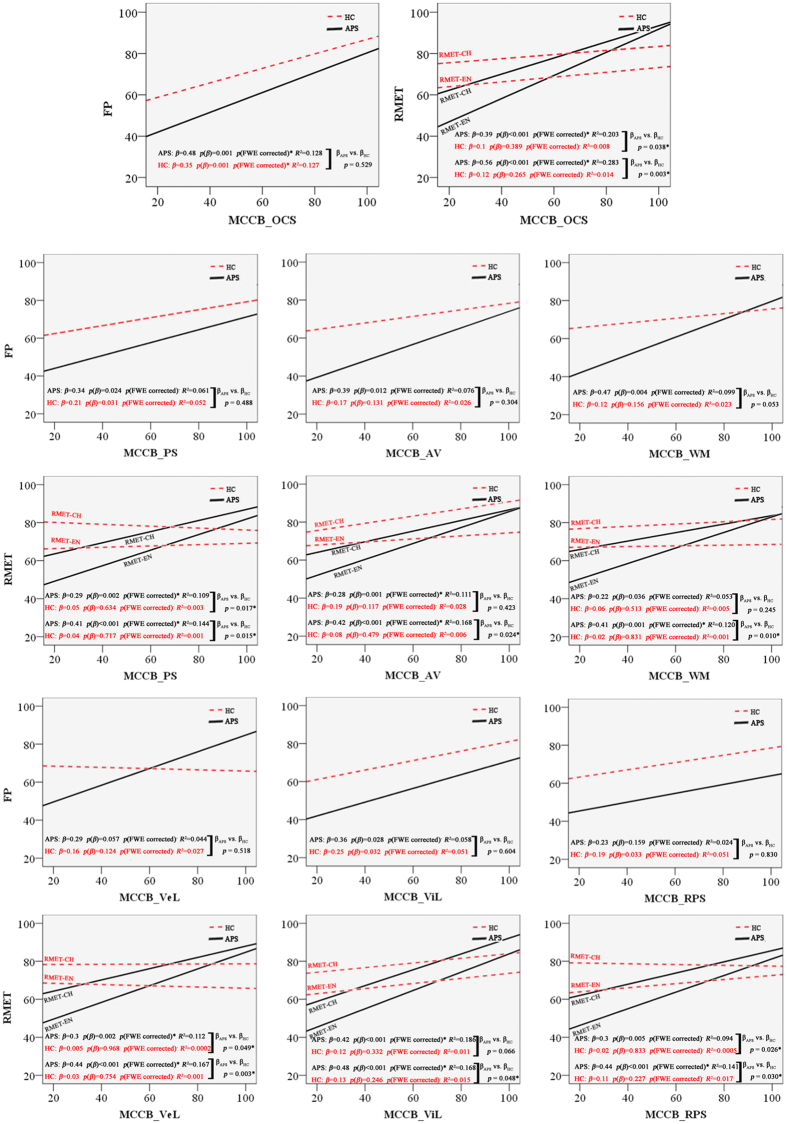
Correlations between FP and RMET performance and MCCB scores in APS (solid line) and HC (dashed line) subjects.

Next, we analyzed whether the increased scores of the MCCB test correlated with ToM performance. MCCB and ToM test scores were positively correlated in APS subjects (Fig. 1). Importantly, the increased neurocognition performances in the APS group correlated with the increased levels of ToM performance. However, among HC subjects, this positive correlation trend was not obvious, and was only observed in some neurocognition domains with the FP test score (see *p*_*β*_ values). As the APS slopes were compared with the HC, the increases (slopes) for APS were significantly more than that for HC between RMET and MCCB scores (see *p*_(*β*, APS vs. HC)_ values). In Table 3, most correlations were significant between the ToM tests and the MCCB scores within the APS group even after controlling the FWE, while no significant correlations were found within the HC group.

The trend line indicates the correlation between FP and RMET performance and MCCB scores. The solid trend line illustrates the correlation for the APS data and the dashed line illustrates the correlation for the HC data. Compared with the solid trend line, the slopes of dashed lines declined for all domains, which were especially significant between RMET and MCCB scores. A linear of the fit of the data is included, indicating the trend of the correlation between MCCB domains and FP and RMET tests.

### Predictive value of combining ToM and neurocognition index on APS conversion

In the APS cohort, 78 subjects (94.0%) completed the follow-up at 1 year. Of these individuals, 20 subjects (25.6%) transitioned to a psychotic disorder over the course of follow-up. These converters did not significantly differ from non-converters on age (mean ± SD: 19.5 ± 3.3 and 19.2 ± 5.0, Converters vs. Non-converters, respectively) and gender (Male: 50.0% vs. 42.3%, Converters vs. Non-converters, respectively). The exploratory binary logistic regression analysis disclosed the effects in neurocognition (ViL) and ToM sub-domain (FP) as significant predictors of conversion (Table 4).

**Figure 2:**
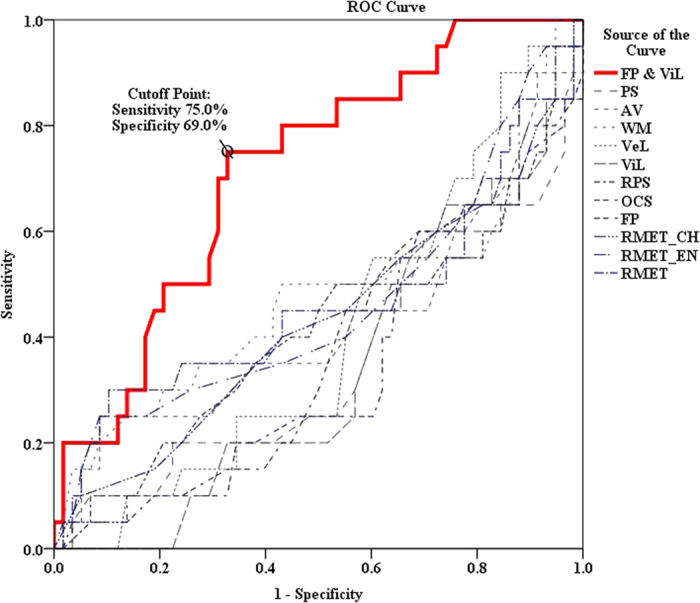
Receiver operating characteristic curves (ROC) of the combined FP-ViL index, compared to individual cognitive domain for predicting psychosis.

**Figure 3:**
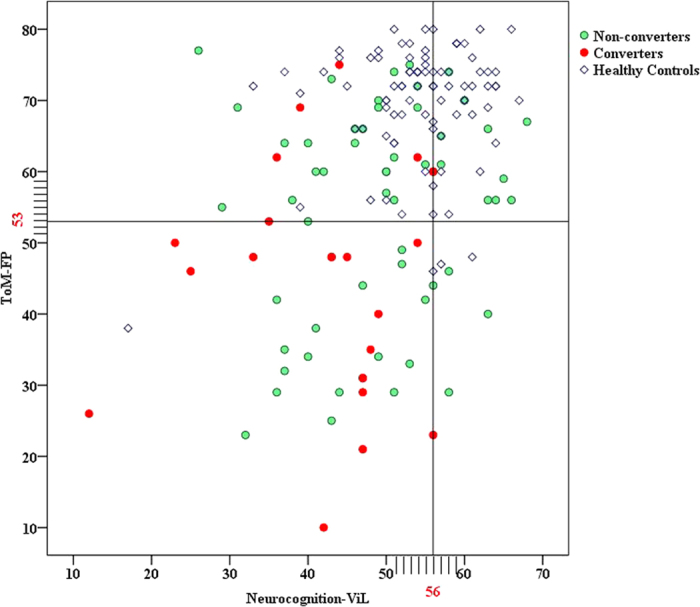
Sensitivity and specificity values of the combined FP-ViL index for predicting psychosis.

Trying to differentiate non-converters from converters using the cognitive measurements as discriminator, using conversion as the principal endpoint (state variable), ROC analysis resulted in an AUC of 0.716 (p = 0.004) for the combined FP-ViL index. However, as for the individual cognitive domain, the AUCs ranged from 0.332 to 0.476 (see Fig. 2.). When non-conversion was used as a state variable, the AUCs ranged from 0.524 to 0.668, and did not reach 0.7. Demanding a sensitivity of 75.0% under moderate specificity (69.0%) for the prediction of psychosis, the combined FP-ViL index cut-off value was FP ≤ 53 and ViL ≤ 56. Figure 3 further illustrates the utility of combined neurocognition and ToM tests in predicting the conversion of psychosis. The combined index was found to have approximately 70.5% accuracy for the APS population (Table 5), which is acceptable for clinical application.

## Discussion

To our knowledge, this was the first study to thoroughly examine whether the ToM deficits found among APS subjects were affected by neurocognitive function, using a relatively large-scale cross-sectional investigation in a Chinese clinical setting. One of the main findings in this study was that ToM ability in the prodromal stage of psychosis was more often associated with neurocognition compared to healthy controls. There is growing evidence supporting the presence of deficits in neurocognition^33^ or social cognition^34^ prior to the onset of full psychosis, which are predictive of later psychosis conversion^35^. Few studies have established this relationship, which could be fundamental for the development of psychosis. Determining the intensity of “attenuate” psychosis is emphasized by the level of conviction of psychotic experience, which also can be considered a critical manifestation of ToM ability. From this point of view, psychosis onset can be accompanied by the gradual conviction of the false beliefs of others’ minds; this is analogous to the decline of ToM ability. One potential interpretation of the data is that if neurocognition can compensate for ToM deficits, it may delay or prevent the onset of psychosis, which would otherwise make an individual more prone to conversion. Therefore, we speculated that psychosis onset underlies the dysfunctional process of both ToM and neurocognition, and that ToM may provide a potential mechanism to link neurocognition to psychotic development^36^.

Further, these data confirm that poor cognitive functioning is associated with a higher risk of conversion to psychosis^37^. Interestingly, the logistic regression model that best explains the risk of conversion indicated that the effects of neurocognition and ToM may be an important factor in psychosis development. Most previous studies revealed that individuals at risk for psychosis possessed deficits in neuropsychological isolated domains such as memory function^5^ and verbal fluency^38^.The results of these studies are difficult to generalize to the new criteria to determine if subjects with deficits in isolated cognitive domains will develop psychosis. Therefore, if ToM deficits accompany abnormal neurocognition function, this association would probably enhance the prediction for psychosis conversion in APS subjects. Although the combined index of ToM and neurocognition proposed in this study only reached reasonable psychometric properties (e.g., 75.0% sensitivity and 69.0% specificity) for differentiating converters from non-converters among APS subjects, it will help to increase the predictive power in this population (e.g., less than 30% APS subjects converting to psychosis)^10^.

As expected, APS subjects performed more poorly on all sub-domains of neurocognition tested by the MCCB. However, most notably, visual learning function at baseline (measured by the Brief Visuospatial Memory Test-Revised, BVMT-R) was found to have significant predictive value for the transition to psychosis. This result is consistent with a recent study conducted on an ultra-high risk population, which clearly confirmed that visuospatial memory deficits were predictive of conversion^39^. Our previous study^5^ also found that only the mean score of the visuospatial domain on the RBANS (Repeatable Battery for the Assessment of Neuropsychological Status) test was significantly lower among the APS subjects compared to controls. Taken together, these results may reflect an important phenomenon which is less emphasized in the literature. Visuospatial functioning deficits have been reported to be related to impairments in self-reflectivity and decentration (e.g., self-other misattribution) in stable schizophrenia patients^40^, which can cause deficits in the inference of others’ intentions (e.g., ToM).

In particular, this study supports the notion that the less significant features of the correlation between ToM and neurocognition are found in DMS processing rather than RMS. However, they are more significant in APS subjects compared to HC subjects. We argue that although both levels of ToM work to capture the mental states of others, in contrast, RMS may involve more complex aspects of ToM, which rely heavily on neurocognition, particularly in terms of interference by psychotic experiences. This improved understanding of the correlation between ToM and neurocognition in prodromal psychosis may allow clinicians to consider how fundamental neurocognition deficits dynamically impact the conviction level of psychosis (e.g., poor judgments about others’ mental states), even leading to withdrawal from social contacts. In other words, when the impaired ToM ability of APS subjects is not sufficient to infer others’ mind states, neurocognition may partially compensate for these difficulties. Combined cognitive remediation^41^, specifically when targeting visuospatial functioning and high-order social cognitive (e.g., RMS) impairments, may be a promising approach to delay or even prevent progression to psychosis.

The most interesting result found in the current models was the differential correlative effect between ToM ability and neurocognition among APS subjects compared to the HC group. Our data from the APS group revealed that neurocognition and ToM performance were significantly related. However, these correlations became less clear in the data from the HC group. Although patients with schizophrenia demonstrated a medium impairment in premorbid general neurocognitive functioning (e.g., IQ)^42^, it still remains unclear whether general intelligence can contribute to the onset of psychosis^43^. Our results suggest that neurocognition may strengthen the skills of APS subjects to make mental state inferences, whereas impaired neurocognition may be less able to compensate for ToM deficits in this pre-onset stage of psychosis. Consequently, APS subjects with impairments in both neurocognition and ToM could experience more misunderstandings regarding the complexities of social interactions^44^, which increases the risk of conversion to psychosis^45^ as well as poor functional outcomes^46^.

In this study, we had a large sample of APS and comparable HC subjects, who were followed up within a reasonable period, and tested by a comprehensive and standardized battery of cognitive measures, with no confounding antipsychotic use and psychotherapy. However, several limitations to our study should be taken into account. First, APS subjects in this study were followed-up only within a 1-year time period, which could limit the number of non-converters who were not classified correctly. Second, this study employed naturalistic observations during the follow-up process; APS was treated with different therapies and medications with different levels of compliance, which may have confounded the results of psychosis conversion. Third, although we restricted APS subjects to the clinical high risk population, this sample presented with considerable heterogeneity in their psychotic symptoms, which could have skewed our results. For example, APS subjects with paranoid symptoms may have performed differently in ToM tasks compared with APS subjects with perceptual abnormalities^47^. Finally, although as expected, MCCB domains more strongly related to ToM tests in APS than in HC, indicative of a compensatory role of neurocognition in ToM functioning, the conclusion cannot be drawn directly. It could be argued that potential factors such as lack of effort might be drivers of the relationship. Future studies are needed with a longer follow-up period and with specific, tailored interventions targeting neurocognitive and neuropsychological difficulties for the APS population.

## Conclusion

Taken together, these data suggest that the way APS subjects infer others’ intentions is likely by neurocognition (e.g., visuospatial functioning). A composite index of neurocognition and ToM could improve the predictive validity of future conversion to psychosis in APS population. These results may lead to future developments of a modified clinical standard for predicting psychosis conversion by combining both neurocognitive and ToM deficits. Additionally, these results are also very important for combined ToM and neurocognitive rehabilitation strategies^47^, which could be a more effective intervention for subjects in the attenuated and pre-onset stages of psychosis.

## Additional Information

**How to cite this article**: Zhang, T.*et al*. Correlation of social cognition and neurocognition on psychotic outcome: a naturalistic follow-up study of subjects with attenuated psychosis syndrome. *Sci. Rep*. **6**, 35017; doi: 10.1038/srep35017 (2016).

## Figures and Tables

**Table 1:** Demographics and clinical variables, comparing APS subjects to healthy controls.

Variables	APS	HC	APS vs. HC	
			*t/χ*^*2*^	*p*
Cases (*n*)	83	90	—	—
Age (years), *Mean (SD)*	19.1 (2.0)	20.3 (1.7)	−1.699	0.091
Male, *n* (%)	48 (57.8)	46 (51.1)	0.786	0.375
Education (years), *Mean* (*SD*)	10.2 (2.0)	10.9 (2.0)	−1.916	0.057
Course (month), *Mean* (*SD*)	5.2(3.5)	—	—	—
SIPS/SOPS				
Family History [*n* (*%*)]	11 (13.3)	0	—	—
Current GAF, [*Mean* (*SD*)]	52.4 (7.2)	80.2 (1.8)	−34.261	<0.001
P1 > 2, Unusual Thought Content, [*n* (*%*)]	59 (71.1)	0	—	—
P2 > 2, Suspiciousness, [*n* (*%*)]	61 (73.5)	0	—	—
P3 > 2, Grandiose Ideas, [*n* (*%*)]	0	0	—	—
P4 > 2, Perceptual Abnormalities, [*n* (*%*)]	50 (60.2)	0	—	—
P5 > 2, Disorganized Communication, [*n* (*%*)]	4 (4.8)	0	—	—
Positive symptoms, [*Mean* (*SD*)]	9.0(2.9)	0.1(0.4)	28.065	<0.001
Negative symptoms, [*Mean* (*SD*)]	14.5(5.7)	0.4(0.7)	22.470	<0.001
Disorganized symptoms, [*Mean* (*SD*)]	6.7(2.9)	0.3(0.5)	19.781	<0.001
General symptoms, [*Mean* (*SD*)]	8.8(3.1)	0.7(1.0)	22.695	<0.001
Total score, [*Mean* (*SD*)]	39.0(9.6)	1.5(1.4)	35.134	<0.001

*Note:*^a^Pearson chi-square with Yates’s continuity correction; ^b^Fisher’s exact test.

**Table 2:** Comparison of the cognitive performances between the APS and HC group.

Variables	APS	HC	*t (p)*	APS	HC	*F (p)*	APS	HC	*F (p)*
	Mean(SE)			Mean(SE) ^a^			Mean(SE) ^b^		
Neuro-Cognition									
SP	43.4	54.1	−6.574	43.3	54.2	43.791	—	—	—
	(1.3)	(1.0)	(<0.001)	(1.2)	(1.1)	(<0.001)			
AV	42.8	49.6	−4.242	43.0	49.5	16.502	—	—	—
	(1.4)	(0.8)	(<0.001)	(1.2)	(1.1)	(<0.001)			
WM	40.7	46.3	−3.487	40.6	46.4	11.949	—	—	—
	(1.2)	(1.1)	(0.001)	(1.2)	(1.1)	(0.001)			
VeL	40.9	47.2	−3.942	40.6	47.4	18.522	—	—	—
	(1.3)	(0.9)	(<0.001)	(1.1)	(1.1)	(<0.001)			
ViL	47.8	54.1	−4.373	47.7	54.3	20.696	—	—	—
	(1.2)	(0.8)	(<0.001)	(1.0)	(1.0)	(<0.001)			
RPS	48.5	53.4	−3.103	48.4	53.6	10.678	—	—	—
	(1.2)	(1.0)	(0.002)	(1.1)	(1.1)	(0.001)			
OCS	41.0	50.7	−6.033	40.9	50.9	38.349	—	—	—
	(1.3)	(0.9)	(<0.001)	(1.2)	(1.1)	(<0.001)			
Social Cognition									
FP	52.0	69.6	−8.782	52.0	69.6	79.490	54.2	67.6	40.527
	(1.8)	(0.9)	(<0.001)	(1.4)	(1.4)	(<0.001)	(1.4)	(1.4)	(<0.001)
FP-F	72.8	90.2	−5.489	72.8	90.2	31.327	75.3	87.9	13.022
	(2.9)	(1.4)	(<0.001)	(2.2)	(2.1)	(<0.001)	(2.4)	(2.2)	(<0.001)
FP-T	68.0	81.3	−4.595	68.4	80.9	18.224	68.5	80.8	13.624
	(2.4)	(1.6)	(<0.001)	(2.1)	(2.0)	(<0.001)	(2.2)	(2.1)	(<0.001)
RMET-CH	70.4	78.4	−5.378	70.6	78.3	25.467	72.0	77.0	9.220
	(1.2)	(0.9)	(<0.001)	(1.1)	(1.0)	(<0.001)	(1.1)	(1.1)	(0.003)
RMET-EN	58.8	67.5	−5.286	59.0	67.3	25.842	61.0	65.5	6.684
	(1.4)	(0.9)	(<0.001)	(1.2)	(1.1)	(<0.001)	(1.2)	(1.1)	(0.011)

*Note:*^a^The means adjusted for age and education were calculated and compared using analyses of covariance; ^b^The means adjusted for age, education, SP, AV, WM, VeL, ViL, RPS and OCS were calculated and compared using analyses of covariance.

Abbreviations: SP: Speed of Processing; AV: Attention/Vigilance; WM: Working Memory; VeL: Verbal Learning; ViL: Visual Learning; RPS: Reasoning and Problem Solving; OCS: Overall Composite Neurocognitive Score; FP: Faux Pas; FP-F: Faux Pas Story; FP-T: No Faux Pas Story; RMET-CH: Reading Mind from Eye test (Asian Version); RMET-EN: Reading Mind from Eye test (Western Version).

**Table 3:** Correlations of ToM tests with MCCB Scores within the APS and HC Groups.

		APS			HC		
		FP	RMET_CH	RMET_EN	FP ^a^	RMET_CH ^a^	RMET_EN
SP	*r*	0.248^*^	0.330^**^	0.380^**^	0.106	−0.113	0.039
	*p*_*r*_	0.024	0.002^#^	0.000^#^	0.322	0.288	0.718
AV	*r*	0.275^*^	0.348^**^	0.418^**^	0.213^*^	0.113	0.076
	*p*_*r*_	0.012	0.001^#^	0.000^#^	0.044	0.290	0.479
WM	*r*	0.315^**^	0.230^*^	0.347^**^	0.126	0.011	0.023
	*p*_*r*_	0.004	0.036	0.001^#^	0.237	0.919	0.831
VeL	*r*	0.210	0.334^**^	.409^**^	0.150	−0.050	−0.034
	*p*_*r*_	0.057	0.002^#^	0.000^#^	0.159	0.640	0.754
ViL	*r*	.242^*^	0.431^**^	0.410^**^	0.046	−0.089	0.124
	*p*_*r*_	0.028	0.000^#^	0.000^#^	0.665	0.404	0.246
RPS	*r*	0.156	0.307^**^	0.375^**^	0.101	−0.085	0.129
	*p*_*r*_	0.159	0.005	0.000^#^	0.344	0.423	0.227
OCS	*r*	0.348^**^	0.452^**^	0.533^**^	0.238^*^	−0.012	0.119
	*p*_*r*_	0.001^#^	0.000^#^	0.000^#^	0.024	0.910	0.265

*Note:*^a^non-parametric Spearman correlations were applied because the FP and RMET-CH scores from the HC dataset did not fit the normal distribution. *p < 0.05, **p < 0.01, ^#^*p* < 0.0024, by controlling the family-wise error (FWE), at the 0.0024 (*p* < 0.05/21) level using a Bonferroni correction.

**Table 4:** Logistic regression for predicting the transition to psychosis (forward stepwise).

Predictor in the Model	Beta	S.E.	Odds Ratio	95% CI	Wald statistic	P value
Cognitive and demographic predictors: SP; AV; WM; VeL; ViL; RPS; FP; RMET; Age; Gender; Education						
FP	−0.068	0.030	0.934	0.881–0.991	5.089	0.024
ViL	−0.039	0.019	0.962	0.927–0.998	4.260	0.039
Education	0.324	0.119	1.382	1.095–1.745	7.432	0.006

**Table 5:** The validity of combined index of FP and ViL (Cutoff point: FP ≤ 53 and ViL ≤ 56).

Sample	*n*	Sensitivity	Specificity	Accuracy	YI	+LR	−LR	+PV	−PV	Kappa
APS	78	75.0%	69.0%	70.5%	0.44	2.4	0.4	45.5%	88.9%	0.4

*Note:* APS: APS subjects completed 1-year follow-up. Abbreviations: YI: Youden’s index; Positive likelihood ratio:+LR; Negative likelihood ratio: −LR; Positive predictive value:+PV; Negative predictive value: −PV.
